# Etiology and clinical features of infection-associated plastic bronchitis in children

**DOI:** 10.1186/s12879-023-08529-w

**Published:** 2023-09-07

**Authors:** Feng Huang, Wenjing Gu, Jianfeng Diwu, Xinxing Zhang, Yanyu He, Youjian Zhang, Zhengrong Chen, Li Huang, Meijuan Wang, Heting Dong, Shanshan Wang, Yuqing Wang, Canhong Zhu, Chuangli Hao

**Affiliations:** 1 Department of Respiration, Children’s Hospital of Soochow University, No. 303 Jing De Road, Suzhou, 215003 China; 2 Department of Pediatric, Xunyi County Hospital, Xianyang, 711300 China; 3 Department of Clinical laboratory, Children’s Hospital of Soochow University, Suzhou, 215003 China

**Keywords:** Plastic bronchitis, Infection, *Mycoplasma pneumonia*, *Boca virus*, Electronic bronchoscopy

## Abstract

**Objective:**

To investigate the etiological characteristics of plastic bronchitis (PB) caused by pulmonary infections in children and to identify any differences in the clinical features of PB cases caused by different pathogens.

**Method:**

We collected data on children diagnosed with PB and admitted to the Respiratory Department at Soochow University Children’s Hospital between July 2021 and March 2023 utilizing electronic bronchoscopy. We analyzed clinical characteristics and the species of pathogens causing the illness in these children.

**Result:**

A total of 45 children were enrolled. The main clinical symptoms observed were cough (100%), fever (80%), shortness of breath (28.9%), and wheezing (20.0%). Pathogens were identified in 38 (84.4%) patients. *Mycoplasma pneumoniae* (*MP*) had the highest detection rate at 53.3%, followed by the *Boca virus* at 26.7%. *MP*-induced PB typically occurs in older children with an average age of 7.46 ± 2.36 years, with the main symptoms including high fever (85.7%) and local hyporespiration (42.9%). In contrast, *Boca virus*-induced PB tends to occur in younger children, with the main symptoms of moderate fever (54.5%), and wheezing (54.5%). The *MP* group exhibited a higher incidence of both internal and external pulmonary complications, including pleural effusion (42.9%), elevated aspartate aminotransferase (52.4%), lactic dehydrogenase (76.2%), and D-D dimer (90.5%). Conversely, the *Boca virus* group primarily showed pulmonary imaging of atelectasis (81.8%), with no pleural effusion. The average number of bronchoscopic interventions in the *MP* group was 2.24 ± 0.62, which was significantly higher than that required in the *Boca virus* group (1.55 ± 0.52). During the second bronchoscopy, 57.1% of children in the *MP* group still had visible mucus plugs, while none were observed in the *Boca virus* group.

**Conclusion:**

*MP* and *Boca virus* are the primary pathogens responsible for PB among children. The clinical manifestations of PB typically vary significantly based on the pathogen causing the condition.

## Background

Plastic bronchitis (PB) is a rare, partial or complete airway obstructive disease characterized by the formation of arborising, thick, tenacious casts of the tracheobronchial tree [1]. It occurs in multiple clinical settings and its cause is not completely understood [2]. Most reported cases have been in children and associated with the Fontan operation for congenital heart disease [3]. Additionally, PB has been reported in individuals with asthma [4], pulmonary trauma [5], lung transplantation [6], and acute respiratory viral infections [7]. But so far, the pathogenic spectrum of infection-associated PB and the clinical characteristics of different pathogens are not clear.

Electronic bronchoscopy and bronchoalveolar lavage (BAL) are indispensable techniques for investigating and treating pediatric patients with PB and both are carried out as routine procedures in many health centers [8–10]. In this study, we collected clinical data, laboratory data, and lung imaging from children diagnosed with PB to explore the pathogenic spectrum of PB caused by pulmonary infections in children. We also investigated the differences in clinical features of PB caused by different pathogens.

## Methods

### Patients and specimen collection

A retrospective study was conducted on 45 pediatric patients who were diagnosed with PB through electronic bronchoscopy and admitted to the Respiratory Department of Children’s Hospital of Soochow University from July 2021 to March 2023. Patients with congenital heart disease and sickle-cell disease were excluded from the study.

### Initial evaluation

Upon admission, demographic and clinical information was collected from all patients. Within four hours of admission, venous blood samples were taken for blood routine, liver and kidney function, coagulation routine, and pre-transfusion examination. Chest radiography was performed within 24 h prior to admission or within the next 24 h. All patients underwent electronic bronchoscopy within 72 h after admission and BAL samples were collected. In some cases, electronic bronchoscopy was reviewed 3–4 days after initial examination.

### Electronic bronchoscopy

Before the electronic bronchoscopy procedure, parents were notified of the potential surgical risks and provided informed consent. Pediatric patients were required to fast from both solids and liquids for at least 6–8 h prior to the procedure. Premedication in the form of intramuscular atropine sulfate, at a dose of 0.01–0.02 mg/kg, was given to the children. The procedure was performed under the care of experienced anesthesiologists. An electronic bronchoscopy (Olympus CV260, Tokyo, Japan) was wedged into each lobe. Foreign body forceps or brush were used to clean and remove the plastic sputum suppository in order to restore unobstructed airway passage Normal saline (0.9%) was used for local irrigation before and after cleaning up the plastic phlegm suppository. Local irrigations were 1ml/kg each time, and first 3 lavages were collected by a sterile sputum-collecting pipe (Falcon 50 ml, Becton-Dickinson, Rutherford, NJ, USA). The collected BAL sample was used for cell counts, viral analysis, and microbiological analysis (bacteria and *MP*).

### Cell counts

Differential cell counts were obtained using a modified version of Wright–Giemsa staining (Wright–Giemsa Stain, Baso Diagnostics Inc., Taiwan, China). At least 500 cells were examined for each specimen. The ratios of various cell types in total cell counts were reported.

### Microbiological analysis

BAL samples from plastic bronchitis were tested for 13 types of viruses and bacteria, as well as *Mycoplasma Pneumoniae *(*MP)*. Bacteria were tested by inoculating BAL samples onto blood plates and examining them after incubation for 18–20 h. Bacterial growth > 10^3^ colony-forming units/ml was considered significant. *MP*, *Chlamydia pneumonia* and viruses, including *respiratory syncytial virus*, *adenovirus*, *influenza virus* (A, B, H1N1, H3N2), *parainfluenza virus*, *Boca virus*, *human rhinal virus*, *human coronavirus*, *human metapneumovirus* were investigated by polymerase chain reaction using a 13 Respiratory Pathogen Multiplex Detection Kits (PCR Capillary Electrophoresis Fragment Analysis) (Hailshi Gene Technology Co., Ltd, Ningbo, China) according to the manufacturer’s instructions.

### Statistical analysis

Measurement data were presented as the mean ± standard deviation (SD). Counting data is expressed as a percentage. Comparison between groups were performed by t test, or Fisher’s exact probability method. A *P* value < 0.05 was considered statistically significant.

## Results

### Demographic information

Between July 2021 and March 2023, our department diagnosed 45 cases of PB. The oldest patient was 12 years and 8 months old, while the youngest was 1 year and 3 months old. The average age of the patients was (5.63 ± 2.94) years old, with 28 (62.2%) being male and 17 (37.8%) being female.

### Electronic bronchoscopy findings

All patients undergoing electronic bronchoscopy showed blockage of the lumen by mucus sputum plugs. The plastic sputum plugs obstructing the bronchus were successfully removed after cleaning with foreign body forceps and brush. The plastic phlegm products were found to be located in the left bronchus or its branches in 22 cases (48.9%), in the right bronchus or its branches in 20 cases (44.4%), and in both in 3 cases (6.7%).

The BAL sample analysis revealed neutrophilic inflammation in PB. The mean percentage of neutrophils was (67.00 ± 22.58)%. Eosinophil levels were elevated in only two patients, with 6% present in one case and 7% in the other.

### Clinical features

All 45 cases (100%) presented with cough symptoms. Additionally, 36 cases (80%) had a fever, with 1 case experiencing low fever (37.3℃-38.0℃), 9 cases with moderate fever (38.1℃-39.0℃), and 26 cases with high fever (39.1℃-41.0℃). Wheezing was observed in 9 cases (20.0%), shortness of breath in 13 cases (28.9%), and the three depressions sign in 9 cases (20.0%).

Pulmonary signs: 14 cases (31.1%) had reduced breath sounds on auscultation in a localized area. 15 cases (33.3%) had wheezing sounds. 10 cases (22.2%) had wet rales. And in 10 cases (22.2%) no positive pulmonary signs were identified.

### Image characteristic

All children exhibited a large flake shadow on their lung imaging. Out of them, 21 patients (46.7%) displayed atelectasis, 12 patients (26.7%) had pleural effusion, 1 patient (2.2%) exhibited pneumothorax, and 1 patient (2.2%) had bronchial expansion as a complication.

### Etiological detection

All patients underwent testing to determine the etiology of the BAL sample, with a specific pathogen identified in 38 cases (84.4%), while the remaining 7 cases yielded negative results. Among the children with a positive pathogen identification, 24 (53.3%) tested positive for *MP*, 12 (26.6%) tested positive for *Boca virus*, 2 (4.4%) tested positive for *Influenza A*, and 1 (2.2%) tested positive for *human rhinovirus*. Of these cases, 2 were concurrently diagnosed with both *MP* and *Streptococcus pneumoniae* infections, 1 with *MP* and *Boca virus* infections, and 1 with *Influenza A* and *Streptococcus pneumoniae* infections.

### Comparison of clinical features of Mycoplasma Pneumonia and Boca virus induced plastic bronchitis

**Fig. 1:**
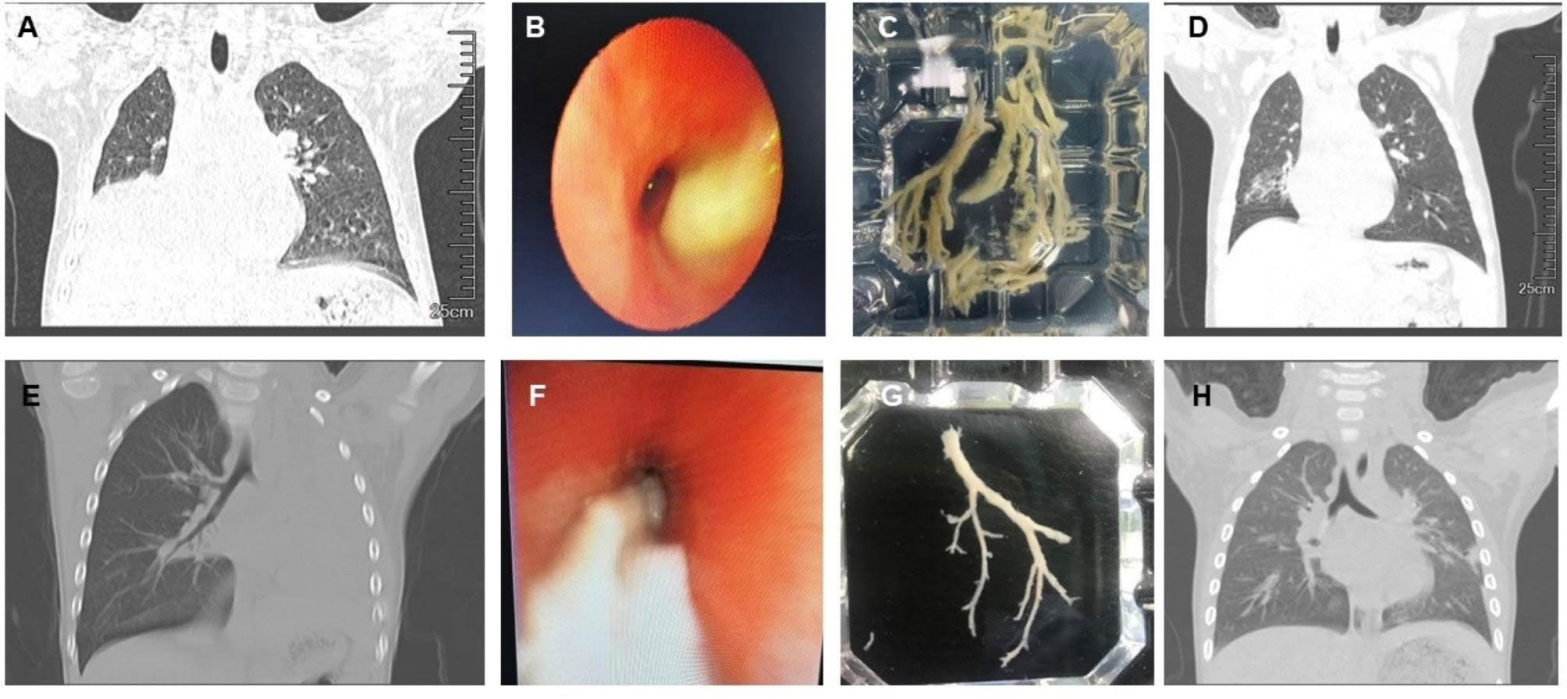
The relevant radiological and bronchoscopy images. **Images A-D represent a 5-year-4-month-old girl diagnosed with MP Infection.** A: Chest CT on admission showed large consolidated inflammation in the right lung, accompanied by atelectasis of the right lower lobe; B: Bronchoscopy revealed mucus plug obstructing the right lower lobe bronchus; C: The mucus plug was successfully removed; D: One month later, a follow-up chest CT showed almost complete resolution of the lung inflammation and complete lung re-expansion. **Images E-H represent a 3-year-5-month-old boy diagnosed with Boca virus infection.** E: Chest CT on admission showed high-density shadows and atelectasis in the left lung; F: Bronchoscopy revealed mucus plug obstructing the left main bronchus; G: The mucus plug was successfully removed; H: Eight days later, a follow-up chest CT showed complete lung re-expansion and almost complete resolution of the inflammation in the left lung

For comparative analysis, we included only children infected with either *MP* or *Boca virus* alone. Data specifically related to these groups can be found in Table 1. The relevant radiological and bronchoscopy images are shown in Fig. 1. The mean age of children in the *Boca virus* group was significantly lower than that in the *MP* group. This difference was found to be statistically significant (*P* < 0.001). There was no significant difference in sex ratio between the two groups (*P* = 0.712). The average length of hospital stay for the *MP* group (11.62 ± 3.12 days) was slightly longer than that for the *Boca virus* group (10.18 ± 3.49 days), but the difference was not statistically significant (*P* = 0.244).

**Table 1:** Clinical features of *Mycoplasma Pneumonia* and *Boca virus* induced plastic bronchitis

		*Mycoplama Pneumonia* n = 21	*Boca Virus* n = 11	*t*/*t’*/Fisher	*P*
**Age**		7.46 ± 2.36	2.86 ± 1.07	6.107	**< 0.001**
**Sex**		11/10	7/4	——	0.712
**Hospital stay**		11.62 ± 3.12	10.18 ± 3.49	1.189	0.244
**Clinal manifestation**	**Cough**	21 (100%)	11 (100%)	——	——
	**Fever**	21 (100%)	10 (90.9%)	——	0.344
	low fever	1 (4.8%)	0 (0%)	——	1.000
	moderate fever	2 (9.5%)	6 (54.5%)	——	**0.010**
	high fever	18 (85.7%)	4 (36.3%)	——	**0.013**
	**Wheezing**	0 (0%)	6 (54.5%)	——	**0.001**
	**Tachypnea**	4 (19.0%)	5 (45.5%)	——	0.213
**Physical signs**	**three depressions sign**	1 (4.8%)	5 (45.5%)	——	**0.011**
	**Pulmonary rales**				
	moist rale	5 (23.8%)	3 (27.3%)	——	1.000
	Wheezing rales	0 (0%)	8 (72.7%)	——	**< 0.001**
	hyporespiration	9 (42.9%)	2 (18.2%)	——	0.248
	non apparent abnormality	7 (33.3%)	1 (9.1%)	——	0.209
**Lung Imaging**	Large shadow	21 (100%)	11 (100%)	——	——
	atelectasis	8 (38.1%)	9 (81.8%)	——	**0.028**
	pleural effusion	9 (42.9%)	0 (0%)	——	**0.013**
	pneumothorax	0 (0%)	1 (14.3%)	——	0.344
**Electronic bronchoscopy**	**Molding site**				
	Left airway or its branches	8 (38.1%)	6 (54.5%)	——	0.465
	right airway or its branches	11 (52.4%)	5 (45.5%)	——	1.000
	bilateral	2 (9.5%)	0 (0%)	——	0.534
	**BAL sample cytological classification**				
	Neutrophil	77.10 ± 16.14	56.44 ± 19.62	2.984	**0.006**
	Lymphocyte	9.00 ± 11.58	3.11 ± 2.71	2.147	**0.043**
	phagocyte	13.85 ± 15.23	39.00 ± 21.44	-3.621	**0.001**
**blood routine**	White blood cell ×10^9^/L	7.64 ± 2.23	9.65 ± 3.95	-1.558	0.142
	Neutrophil ×10^9^/L	5.65 ± 1.75	5.47 ± 3.38	0.165	0.871
	Neutrophil%	74.48 ± 10.50	54.04 ± 20.81	3.061	**0.009**
	Lymphocyte ×10^9^/L	1.35 ± 0.83	3.18 ± 2.07	-2.811	**0.016**
	Lymphocyte%	17.24 ± 8.97	35.77 ± 17.09	-3.363	**0.005**
	Eosinophils%>4%	0 (0%)	4 (36.4%)	——	**0.009**
	platelet ×10^9^/L	261.14 ± 108.25	313.55 ± 92.28	-1.364	0.183
**Other blood tests**	ALT>30U/L	5 (23.8%)	0 (0%)	——	0.138
	AST>44U/L	11 (52.4%)	0 (0%)	——	**0.005**
	LDH>382U/L	16 (76.2%)	6 (54.5%)	——	0.252
	D-D dimer>550ug/L	19 (90.5%)	4 (36.4%)	——	**0.003**
**The number of Electronic tracheoscopic interventions**		2.24 ± 0.62	1.55 ± 0.52	3.140	**0.004**
**PB still exist for the second time of Electronic tracheoscope**		12 (57.1%)	0 (0%)	——	**0.002**

Abbreviation: ALT: glutamic-pyruvic transaminase; AST: aspartic transaminase; LDH: lactic dehydrogenase

#### Symptom

All the children in the *MP* group exhibited fever, primarily high fever, accounting for 85.7%, whereas most of the children in the *Boca virus* group experienced moderate fever (54.5%). There was a statistically significant difference in the peak temperature between the two groups (*P* = 0.010). Wheezing was observed in 54.5% of children in the *Boca virus* group, but none in the *MP* group, and there was a statistically significant difference between the two groups (*P* = 0.001). Tachypnea was present in 45.5% of children in the *Boca virus* group, marginally higher than in the *MP* group (19.0%), although the difference was not statistically significant (*P* = 0.213). Similarly, 45.5% of children in the *Boca virus* group exhibited three depressions sign, which was significantly higher compared to 4.8% in the *MP* group (*P* = 0.011).

#### Sign

During pulmonary auscultation, palpable wheezing sounds were present in 72.7% of children in the *Boca virus* group, whereas none in the *MP* group exhibited wheezing sounds. This difference was statistically significant (*P* < 0.001). Conversely, 42.9% of the children in the *MP* group displayed local respiratory hypotonia, which was slightly higher compared to the *Boca virus* group, though the difference was not significant (P = 0.248).

#### Lung imaging

The prevalence of atelectasis was higher in children in the *Boca virus* group (81.8%) compared to those in the *MP* group (38.1%). Conversely, children in the *MP* group had a higher likelihood of developing pleural effusion (42.9%), while none exhibited pleural effusion in the *Boca virus* group. These differences were statistically significant (*P* = 0.028, 0.013).

#### Electronic bronchoscopy

There was no statistical discrepancy between the two groups in terms of the formation site of plastic phlegm suppository (*P* > 0.05). The percentage of neutrophils and lymphocytes in the *MP* group was considerably higher than that in the *Boca virus* group (*P* = 0.006, 0.043). The proportion of phagocytic cells in the *Boca virus* group was significantly greater than that in the *MP* group (*P* = 0.001).

#### Laboratory examination

There were no significant discrepancies in the total number of white blood cells, absolute value of neutrophils, and platelet count between the two groups. The percentage of neutrophils was significantly elevated in the *MP* group (*P* = 0.009), while the percentage and absolute value of lymphocytes were significantly higher in the *Boca virus* group (*P* = 0.005, 0.016). Furthermore, the elevated percentage of eosinophils in children with *Boca virus* was higher than that in the *MP* group (*P* = 0.009). Additionally, the *MP* group was more prone to experiencing other systemic complications. The proportions of increased AST and D-D dimer were significantly higher in this group compared to the *Boca virus* group (*P* = 0.005, 0.003). No elevated aminotransferase was identified in the *Boca virus* group.

#### Prognosis

The average number of bronchoscopic interventions needed in the *MP* group was 2.24 ± 0.62, which was greater than the *Boca virus* group (1.55 ± 0.52), and the difference was statistically significant (*P* = 0.004). During the second bronchoscopy, 57.1% of the children in the *MP* group still had visible mucus plugs, whereas none of the children in the *Boca virus* group had visible mucus plugs (*P* = 0.002). These findings suggest that performing a second tracheoscopy intervention may not be necessary in the *Boca virus* group.

## Disscussion

PB is characterized by the presence of thickened bronchial casts that can be coughed up or detected during bronchoscopy or surgical procedures. The etiology of PB is multifaceted, with infectious pathogens being an important contributing factor. Several pathogens have been reported in the literature as potential causes of PB, including *MP* [7], *influenza virus A* [4], *adenovirus* [11, 12] and *Boca virus* [13]. Our study included 45 children diagnosed with PB, among whom 38 tested positive for pathogens. More than half of the positive cases were associated with *MP* and around 1/4 were associated with *Boca virus*, indicating that these two are the primary pathogens responsible for infection-related PB. Other causative agents include *influenza virus A* and *rhinovirus*.

Plastic bronchitis, due to the formation of sputum in the bronchial cavity, causing severe blockage of the airway, causing clinical symptoms such as shortness of breath and breathing difficulties, which can be life-threatening in severe cases [14, 15]. The clinical manifestations of PB are closely linked to the location and extent of airway obstruction caused by the sputum plug. In our study, approximately 25% of the children experienced shortness of breath and dyspnea. The majority of these cases were associated with blockages in the main bronchus or multiple sputum plugs.

Although both *MP* and *boca virus* can cause PB, there are noticeable differences in their clinical manifestations. Currently, much research has been conducted on PB caused by *MP* [7, 16, 17]. Persistent fever before bronchoscopy, extrapulmonary complications, pleural effusion, cough duration, and LDH levels are all risk factors for bronchoplastic formation of *MP* pneumonia [7]. In our study, we also confirmed that PB caused by *MP* was primarily characterized by high fever. Previous studies have shown that *MP* can also cause wheezing in children [18] However, in this study, none of the children infected with *MP* wheezed, but the local respiratory sound was low. It may be related to the complete blockage of the lumen by the plastic-shaped sputum plug formed by *MP* infection. Studies showed that MUC5AC and MUC5B played an important role in the formation of mucin plugs [19]. The elevated concentrations of MUC5B leads to mucociliary clearance dysfunction and enhances lung fibrosis in mice [20]. Our previous research also showed that the high expression of MUC5AC, MUC5B, and layilin played an essential role in prediction in the development of PB caused by *MP* [21]. This may explain why PB caused by *MP* infection clogs the lumen more firmly, leading to low local breathing sounds rather than wheezing. Of course, we still need more cases to observe in the later stage. There were also more internal and external pulmonary complications in *MP* cases, including pleural effusion, elevated aminotransferase, elevated LDH and D-D dimer levels. The BAL fluid showed significant neutrophilic inflammation.

At present, there is a relative shortage of studies focused on *Boca virus*-induced PB, with most literature limited to case reports [13]. One study revealed that *Boca virus* infected identical twins, leading to the development of PB in both children. This suggests that even a simple *Boca virus* infection can trigger PB in otherwise healthy children [22]. In our study, we further demonstrate that *Boca virus* is a common cause of PB, with a higher incidence in young children, with an average age of 2.86 years. Clinical symptoms typically include moderate fever and wheezing, with nearly all children presenting wheezing sounds in their lungs, with half experiencing difficulty breathing. Pulmonary imaging studies mainly show atelectasis, with no accompanying pleural effusion. As opposed to *MP*, there are fewer extrapulmonary complications in children infected with *Boca virus*, and no elevations in transaminase levels are typically observed.

The treatment for PB typically involves targeted therapies to address the underlying pulmonary condition, as well as interventions to facilitate the removal of mucus plugs. Bronchoscopy is both a diagnostic tool and a primary method of treatment for PB [8]. In this study, electronic bronchoscopy was used to diagnose all children with PB, with some requiring multiple bronchoscopic interventions due to their condition. Children with PB caused by *MP* required significantly more bronchoscopic interventions on average than those caused by *Boca virus*. More than half of the children with *MP* infection still had mucus plugs present during the second bronchoscopic intervention, indicating that a single intervention was often insufficient, whereas those with *Boca virus*-induced PB typically required only one intervention.

## Conclusion

To summarize, infection is a significant contributor to PB in children, presenting as symptoms such as cough, fever, wheezing, and shortness of breath. *MP* and *Boca virus* are the primary pathogens associated with PB in children. There are significant differences in the clinical manifestations of PB caused by different pathogens.

## Data Availability

The datasets used and/or analysed during the current study available from the corresponding author on reasonable request.

## Abbreviations

BAL: bronchoalveolar lavage
*MP*: *Mycoplsma pneumonia*
PB: plastic bronchitis
SD: standard deviation
